# Relationship Between Mediterranean Diet Adherence and Body Composition Parameters in Older Adults from the Mediterranean Region

**DOI:** 10.3390/nu16213598

**Published:** 2024-10-23

**Authors:** Kaja Teraž, Katarina Pus, Saša Pišot, Ana Cikač, Boštjan Šimunič

**Affiliations:** 1 Department of Medical, Surgical, and Health Sciences, University of Trieste, Strada di Fiume 447, 34100 Trieste, Italy; 2 Institute for Kinesiology Research, Science and Research Centre Koper, Garibaldijeva 1, 6000 Koper, Slovenia; 3 Faculty of Sport, University of Ljubljana, Gortanova 22, 1000 Ljubljana, Slovenia; 4 Department of health sciences, Alma Mater Europaea University, Slovenska ulica 17, 2000 Maribor, Slovenia

**Keywords:** nutrition, elderly, body composition, healthy ageing, epidemiology

## Abstract

In recent decades, the rapid spread of various communication media has led to changes in traditional eating habits. In the Mediterranean region, the classic (Mediterranean) dietary pattern has been lost as a result. This has led to a shift in eating habits towards unhealthy eating patterns, which in turn has resulted in an inadequate distribution of body composition. It is known that, among other things, the number of non-communicable diseases increases with the inadequate distribution of body composition. The aim of our study was to examine the level of adherence to the Mediterranean diet (MD) of older adults in the Mediterranean region in relation to specific body composition parameters. This study included 521 older adults with a mean age of 69.6 ± 6.3 years. Body composition was measured using the BIA 101 Anniversary device (Akern s.r.l., Florence, Italy) and adherence to the MD was assessed using the MEDLIFE index questionnaire. This study found significant differences in body composition between males and females. The mean adherence to the MD was 17.0 ± 3.3 points among the participants and there was higher adherence in females (*p* = 0.002, η_p_^2^ = 0.019). A multiple linear regression was performed to assess the relationship between the body composition parameters and MD. Multiple linear regression models were significant for reactance, fat mass (%), fat-free mass (%), skeletal muscle index, and total body water (%), with specific individual MEDLIFE items such as the consumption of processed meat, meat, white meat, fruit, vegetables, olive oil and limiting snacks between meals. Moreover, promising correlations were found between certain MD characteristics and BIA parameters, but the overall health effects of the MD remain unclear.

## 1. Introduction

In recent decades, due to the exponential spread of multiple communication media, modifications of traditional dietary habits have taken place [1]. An unbalanced diet combined with a lack of physical activity can lead to high blood pressure, elevated blood sugar, increased blood lipids, and obesity. All these abnormalities are referred to as metabolic risk factors, which can contribute significantly to the development of non-communicable diseases (NCDs) [2]. NCDs are one of the main causes of death worldwide, accounting for around 75% of deaths in 2019 [3]. NCDs such as cardiovascular disease, cancers, diabetes, chronic respiratory disease, and obesity are a cause of premature death globally [4]. It is known that the incidence of NCDs increases with an inadequate body composition; a higher proportion of adipose tissue leads to obesity [5], insulin resistance [6], type 2 diabetes [7], dyslipidaemia [6], and other metabolic diseases [7]. Nevertheless, the health risk in older adults cannot be assessed merely by considering body fat or fat distribution. For example, reduced muscle mass can lead to reduced muscle strength and, therefore, sarcopenia [8,9]. In comparison to younger individuals, older adults have less muscle and bone mass, increased extracellular fluid volumes, and reduced body cell mass. The fat-free components of body composition are crucial to influencing health in older adults [10]. Specific body composition components can be influenced by diet [11,12,13,14,15]. Nutrition is an important element of a lifestyle that helps one maintain the best possible personal health. An extensive body of evidence substantiates the hypothesis that diet and nutritional factors play a crucial role in the onset of disease [16]. One of the most intensively studied diets is the Mediterranean diet (MD), which is associated with a reduced risk of cardiovascular disease, type 2 diabetes, cancer, and other NCDs [16,17,18,19,20,21].

There are differences between the definitions of the MD, though we can see some similarities among them. The MD emphasises minimally processed foods and includes a high intake of healthy fats, especially extra virgin olive oil, low-glycaemic-index carbohydrates, whole-grain cereals, fruits, legumes, vegetables, and nuts. It also involves the moderate consumption of fish, poultry, dairy products, and red wine with meals, while limiting the intake of red and processed meat, sweets, and sugar-sweetened beverages [18,19,20]. The specific elements of the MD that contribute to obesity prevention and body mass management are the abundance of polyphenols found in extra virgin olive oil, fruits, vegetables, herbs, spices, whole grains, legumes, and nuts. Additionally, this diet features a high level of monounsaturated fatty acids (MUFAs) and a favourable polyunsaturated-to-saturated fatty acid ratio (PUFA/SFA) from extra virgin olive oil, nuts, fish, and seafood. These components possess anti-inflammatory and antioxidant properties that help reduce the chronic inflammation associated with obesity [18]. Furthermore, higher adherence to the MD is related to a lower body mass index in older adults [18]; the high fibre content of vegetables, fruits, whole grains, legumes, and nuts can enhance appetite control by slowing gastric emptying and increasing the feeling of fullness. In addition, polyphenols, *n*-3 polyunsaturated fatty acids (PUFAs), and fibre have a prebiotic effect, which may benefit the altered gut microbiota seen in individuals with obesity compared to those with a normal body mass [18]. Another important aspect of the MD and Mediterranean lifestyle is that people do not consume nutrients and foods in isolation; they eat following specific MD dietary patterns. These patterns are expected to have a wider and more varied impact on biological and behavioural processes. Thus, they are considered more predictive of overall health status and disease risk than focusing on individual foods or nutrients. Moreover, a more sustainable lifestyle is thought to be achieved by changing the entire dietary pattern rather than by simply adding or removing specific foods or nutrients [21].

The MD is linked to positive effects on the occurrence of comorbidities, including metabolic syndrome, hypertension, cardiovascular disease, obesity, cancer, poor bone mineral density, rheumatoid arthritis, and neurodegenerative disorders [22]. Previous research [23,24,25,26] has shown that the development of NCDs is related to the amount of specific body tissues a person has. Moreover, there appears to be a declining trend in adherence to the MD in several Mediterranean countries [1]. Therefore, we aimed to explore the level of adherence to the MD and its relationship with selected body composition parameters in older adults from the Mediterranean basin.

## 2. Materials and Methods

### 2.1. Design and Study Sample

In this cross-sectional observational study, older adults from 11 different Slovenian regions were examined. Measurements were taken between February 2022 and December 2023. The study was conducted in accordance with the Declaration of Helsinki and approved by the National Ethics Committee (0120-76/2021/6). Written informed consent was obtained from all the study participants.

**Figure 1:**
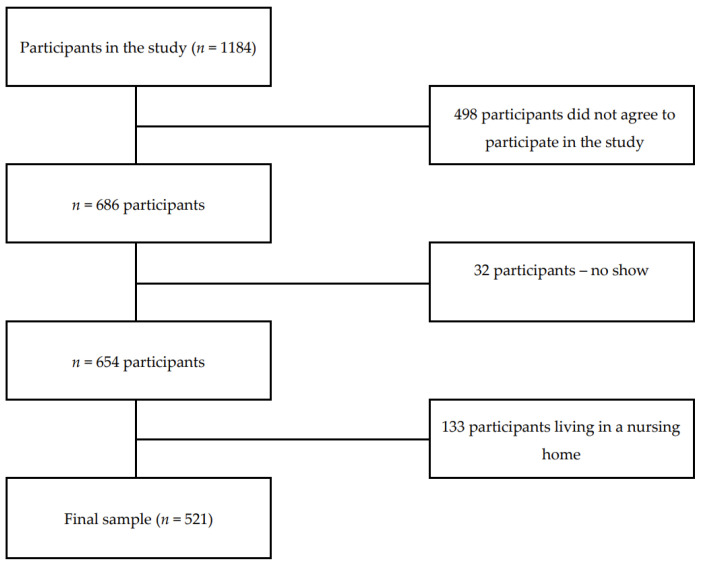
Flowchart of participants included in the present analysis.

Of the 1184 signed consents, 686 agreed to participate in the study, and, of those, 654 older adults (30.4% male) with a mean age of 72.45 ± 8.74 completed the testing. The only inclusion criterium was a minimum age of 60 years; the exclusion criteria were acute illness, exhaustion, terminal cancer, infection, and hospitalisation. Moreover, for this study, all older adults who were living in a nursing home were excluded from the analysis. The final dataset included 521 independently living older adults with a mean age of 69.6 ± 6.3 years (31.1% male) (Figure 1).

### 2.2. Data Collection

The participants were invited to take part in the measurements via regional health centres, at the invitation of a selected general practitioner. The measurements included anthropometric and body composition measurements, as well as questionnaires with information on sociodemographic data and the Mediterranean lifestyle.

Anthropometric and body composition assessment. Body mass (BM) was measured to the nearest 0.1 kg using a scale (Seca 709, Hamburg, Germany), with participants wearing only light clothing and no shoes. Body height (BH) was measured to the nearest 0.5 cm on a standardised height board. The body mass index (BMI) was calculated as the BM (kg) divided by the square of the BH (m). Tetrapolar bioelectrical impedance (BIA 101 Anniversary; Akern-Srl, Florence, Italy) was used to determine body composition in the supine position. The measurement was performed after the participants had been supine for at least 15 min to minimise distortions in body water compartments [27]. The resistance (Rz, Ω), reactance (Xc, Ω), muscle mass (MM, %), skeletal muscle mass index (SMI), fat mass (FM, %), fat-free mass (FFM, %), total body water (TBW, %), extra- and intracellular body water (ECW, ICW, %), phase angle (PA, °), and standardised phase angle (SPA, °) were extracted for the analysis.

The MEDLIFE index questionnaire (Mediterranean Lifestyle Index) was used to assess Mediterranean lifestyle [28,29]. The MEDLIFE index consists of 28 questions divided into three segments: (a) food consumption, (b) traditional dietary habits, and (c) physical activity, rest, and social contact. The first segment contains 15 items on the frequency of food consumption and the portions offered, the second segment contains 7 items typical of Mediterranean dietary habits, and the third segment contains 6 items on physical activity, afternoon naps, social habits, and sociability. For each question, participants can score 1 or 0, giving a maximum of 28 points for the entire questionnaire. A higher score indicates a stronger adherence to the Mediterranean diet and lifestyle. Due to the geographical characteristics of individual Mediterranean regions (and thus specific differences in nutrition and lifestyle habits) [30], the response criteria for awarding one point were slightly changed (compared to the original questionnaire). For item 7, one point was awarded if the participants stated that they ate at least 1 portion per week. For item 12, one point was awarded if they ate at least two fruits per day. For item 15, a point was awarded if they stated that they ate at least 3 servings per day. For item 26, a point was awarded for less than 2 h. For item 28, one point was awarded if they engaged in weekly physical activity in the company of someone else.

MEDLIFE partial scores were calculated by summing up the individual items, from a specific segment; MEDLIFE partial score 1 was calculated by summing up the items from “food consumption”, partial score 2 was calculated by summing up the items from “traditional dietary habits”, and partial score 3 was calculated by summing up the items from “physical activity, rest, and social contacts”.

### 2.3. Data Analyses

All statistical analyses were performed with the statistical software IBM SPSS 27.0 (IBM Corp, Armonk, NY, USA). All parameters are described using mean values and standard deviation (SD). The normality of the data’s distribution was checked and confirmed both graphically (histogram and QQ plots) and analytically (Kolmogorov–Smirnov test). Sex differences in general characteristics and MEDLIFE items were confirmed by a multivariate analysis of variance (MANOVA). To compare answers to individual items of the MEDLIFE questionnaire, a Chi-square test was used. A one-way MANOVA was conducted to compare the total MEDLIFE index and different MEDLIFE partial scores between sexes.

To analyse the adherence to the MD, we summed up partial scores 1 and 2 and calculated their z-values. Participants were then divided into quartiles according to z-values, with quartile 1 reflecting lowest adherence to the MD and quartile 4 reflecting highest adherence to the MD. A one-way ANCOVA was conducted to compare adherence to the MD and BIA parameters, with sex as a covariate. Levene’s test and normality checks were carried out and the assumptions were met. Furthermore, a multiple regression analysis was performed to predict body composition parameters (Rz, Xc, %FFM, %FM, %MM, SMI, %TBW, %ECW, %ICW, PA, and SPA) from all the items of the questionnaire, separately for females and males. All significant decisions were confirmed by *p* < 0.05.

## 3. Results

The general characteristics of the older adults who participated in this study are described in Table 1. The mean age of the participants was 69.6 ± 6.3 years (31.1% male). Differences between sexes were found in selected variables (age, BH, BM, ITM, FM, FFM, MM, SMI, TBW, ECW, ICW, Rz, Xc, PA, SPA, and MEDLIFE score): F (14, 480) = 171.886, *p* < 0.001, Wilks’ Lambda = 0.166, η_p_^2^ = 0.834.

After the Bonferroni correction, males had a higher BH, higher FFM, MM, TBW, and ICW percentages, and a higher PA. On the other hand, as expected, females had a higher proportion of FM, Xc, Rz, SPA, and MEDLIFE. Females also had a higher proportion of extracellular water.

In addition, there were sex differences in the MEDLIFE total scores and MEDLIFE partial scores (F(3, 512) = 3.312, *p* = 0.020, Wilks’ Lambda = 0.981, η_p_^2^ = 0.019) (Table 2). Females scored higher in the MEDLIFE total score, and the MEDLIFE partial score 1, but not in the second and third partial scores.

There was no difference between the sexes in the reported answers to individual items ($\chi^{2}=1.281;\;p=0.258)$.

Table 3 shows the difference in BIA parameters between different levels of adherence to the MD, controlled for sex: FM (F(3, 503) = 3.491; *p* = 0.016; η_p_^2^ = 0.020), FFM (F(3, 503) = 3.491; *p* = 0.016; η_p_^2^ = 0.020), MM (F(3, 503) = 3.601; *p* = 0.013; η_p_^2^ = 0.021), and TBW (F(3, 503) = 4.055; *p* = 0.007; η_p_^2^ = 0.024), which were not statistically significant after adjusting for multiple comparisons.

A multiple regression was run to predict BIA parameters from the MEDLIFE questionnaire, which was successful only for females. The specific coefficients predicting the female BIA parameters from the MEDLIFE questionnaire are shown in Table 4. The Rz model (F (3, 343) = 5.775; R = 0.219; R^2^ = 0.040; *p* < 0.001) and SMI model (F (3, 343) = 5.417; R = 0.213; R^2^ = 0.037; *p* = 0.001) were predicted from the consumption of red meat, white meat, and fruit. The FM (%) model (F (3, 343) = 7.623; R = 0.250; R^2^ = 0.063; *p* < 0.001) and/or FFM (%) model (F (3, 343) = 7.623; R = 0.250; R^2^ = 0.063; *p* < 0.001) were predicted from the consumption of processed meat, olive oil, and snacks between meals. As FFM is derived directly from FM, the FFM model contains the same values as FM, but with a negative sign. The TBW (%) model (F (3, 343) = 7.718; R = 0.251; R^2^ = 0.063; *p* < 0.001) was predicted from the consumption of processed meat, olive oil, and cereals.

## 4. Discussion

This observational study investigated the relationship between MD adherence and selected body composition parameters in older adults from the Mediterranean region. The mean overall MEDLIFE index of the population was 17.0 ± 3.3 points and the results show that females had a higher overall adherence to the MEDLIFE index and collected higher scores in segment “a—food consumption”. As expected, differences in body composition between the sexes were found; males were taller and had higher fat-free mass and muscle mass percentages, total body water, intracellular water, and phase angles. On the other hand, as expected, females had higher fat mass percentages, higher values of reactance, resistance, and SPA, and higher percentages of extracellular water.

The MD is vital for enhancing health and extending the lifespan of older adults [31], and the development of chronic non-communicable diseases is associated with the quality of certain body tissues. The mean overall adherence to the MD of the study population was higher in comparison to the Spanish population [31]. Moreover, a higher adherence to the MD in females was also found in some of the literature [32], though not all studies agree with that [1].

For our females, consuming more than two portions per week of red meat predicted a lower SMI. On the other hand, a sufficient intake of white meat and fruit predicted a higher SMI. When comparing different levels of adherence to the MD, older adults with a higher adherence to the MD had a higher percentage of MM in comparison to those with lowest adherence. The amount of muscle mass retained in older age is important, as a higher muscle mass is associated with lower cardiovascular disease in both sexes. Nevertheless, Srikanthan et al., 2021 [26] highlighted the importance of sexual dimorphism in cardiovascular disease risk and fat mass; in females, gaining muscle mass, and not necessarily losing fat mass, is important in the prevention against cardiovascular diseases.

Additional analyses of females have shown that a higher consumption of processed meat, olive oil, and snacks between meals is associated with a higher percentage of FM. Excess fat mass is related to an increased risk of mortality [33] and mobility-related disability in older adults [34]. Moreover, a higher percentage of FM is associated with an accelerated loss of lean mass and muscle quality [35], which has further implications, as discussed below.

Regarding FFM, typical MD habits such as a lower amount of processed meat, higher consumption of olive oil, and limiting snacking between meals are associated with a higher % of FFM. It has already been established that a greater amount of lean tissue has a protective association with all-cause [33,36] and cardiovascular mortality [36], sarcopenia, and frailty [37]. A higher amount of red meat, along with the recommended amount of white meat and fruit, shows associations with a higher SMI.

The recommended intake (according to the MD) of processed meat and olive oil, along with a lower intake of cereals, shows an association with a higher amount of TBW. Maintaining a proper water balance is essential for muscle function in older adults [38,39].

It is known that an increased consumption of certain food groups such as fruit and vegetables, fish and seafood, and wholemeal cereal products, while limiting the consumption of processed foods (food consumption typical of the Mediterranean region), has a positive effect on non-communicable chronic diseases (NCDs). Rz as a BIA parameter is important for assessing body composition, hydration status, and muscle health in older adults, contributing to overall health monitoring and disease prevention [40]; a higher Rz was associated with the recommended consumption of red meat and a lower consumption of white meat and fruit. In the past decade, Mediterranean populations have exhibited a low-to-moderate adherence to the MD. As a result, there is an urgent need to boost adherence among younger and older adults, as well as among men and women, even in the diet’s countries of origin [1].

Despite the strengths of this study, some limitations should be considered. A major limitation is the lack of detailed information on comorbidities, which may influence other health outcomes and affect the interpretation of the results. In addition, without information on comorbidities, there is a risk that this study may attribute body composition parameters solely to dietary habits, while they may in fact be due to the presence of a chronic disease. Information about comorbidities could have provided more concrete results regarding the role of the MD in the lives of older adults, allowing for a clearer understanding of its impact in the presence of comorbidities. Second, the more precise and accurate collection of data on MD-specific food consumption would provide valuable insight into the distinct roles of individual dietary components in the body composition of older adults. Third, this study only used sex as a covariate when analysing the results. With additional covariates, the model and conclusions would have been more robust.

The main strength of this study is that it focuses on older adults in the Mediterranean region, where the MD is naturally followed. This provides a realistic view of the relationship between diet and body composition. Future research could investigate the long-term effects of the MD on the body composition of older adults, its interactions with certain comorbidities, gender differences, the combined effect of diet and physical activity, regional differences within the Mediterranean region, the influence of social and lifestyle factors, and the specific contributions of individual dietary components to the maintenance of muscle mass and the control of fat distribution.

## 5. Conclusions

The results of this study show a sex difference in the level of adherence to the MD; females were more likely to adhere to the MD. In addition, further analyses showed promising associations between specific MD characteristics and selected BIA parameters. The precise impact of the MD on health is still unclear. To draw more robust conclusions about the relationship between the MD and health status in older adults, intervention studies should be developed further by analysing dietary intake in combination with comorbidities in more detail and in a larger cohort. However, it is believed that the overall dietary pattern, rather than any specific food, contributes to the health of older adults.

## Figures and Tables

**Table 1:** General characteristics of the study participants.

	Total (N = 521)	Male (N = 162)	Female (N = 359)	*p*-Value (η_p_^2^)
Age (years)	69.6 ± 6.3	70.8 ± 6.5	69.1 ± 6.1	0.007 (0.015)
Body height (cm)	164.5 ± 8.1	172.4 ± 6.4	161.0 ± 6.1	<0.001 (0.428)
Body mass (kg)	77.8 ± 14.2	76.3 ± 14.1	78.4 ± 14.1	
Body mass index (kg/m^2^)	28.7 ± 4.7	28.6 ± 4.7	28.7 ± 4.7	
Fat mass (%)	31.0 ± 8.3	25.0 ± 6.8	33.7 ± 7.4	<0.001(0.232)
Fat-free mass (%)	69.0 ± 8.3	75.0 ± 6.8	66.3 ± 7.4	<0.001 (0.232)
Muscle mass (%)	31.2 ± 8.0	37.6 ± 7.8	28.2 ± 6.1	<0.001 (0.287)
SMI	7.9 ± 1.6	9.6 ± 1.2	7.1 ± 1.0	<0.001 (0.536)
TBW (%)	50.6 ± 6.1	55.2 ± 5.0	48.5 ± 5.3	<0.001 (0.254)
ECW (%)	47.2 ± 4.1	46.2 ± 4.5	47.7 ± 3.5	<0.001 (0.029)
ICW (%)	52.8 ± 4.1	53.8 ± 4.5	52.3 ± 3.8	<0.001 (0.029)
Rz(Ω)	551.7 ± 80.1	490.5 ± 63.8	579.7 ± 70.6	<0.001 (0.274)
Xc (Ω)	55.2 ± 9.5	51.0 ± 8.7	57.0 ± 9.3	<0.001 (0.085)
PA (°)	5.7 ± 1.0	6.0 ± 1.1	5.6 ± 0.9	<0.001 (0.025)
SPA (°)	1.2 ± 1.6	0.5 ± 1.5	1.5 ± 1.6	<0.001 (0.066)
MEDLIFE index (pt)	17.0 ± 3.3	16.4 ± 3.4	17.3 ± 3.2	0.002 (0.019)

Bonferroni correction *p* < 0.003; SMI—skeletal muscle mass index, TBW—total body water, ECW—extracellular water, ICW—intracellular water, Rz—resistance, Xc—reactance, PA—phase angle, SPA—standardised phase angle.

**Table 2:** Total and partial scores of the MEDLIFE index for females and males.

	Total (N = 521)	Male (N = 162)	Female (N = 359)	*p*-Value (η_p_^2^)
MEDLIFE total score (pt)	17.1 ± 3.3	16.4 ± 3.4	17.3 ± 3.2	0.002 (0.018)
MEDLIFE partial score 1 (pt)	8.7 ± 2.4	8.3 ± 2.4	8.9 ± 2.3	0.004 (0.016)
MEDLIFE partial score 2 (pt)	4.2 ± 1.4	4.0 ± 1.5	4.3 ± 1.3	0.062
MEDLIFE partial score 3 (pt)	4.0 ± 1.2	3.9 ± 1.2	4.0 ± 1.2	0.353

Bonferroni correction *p* < 0.0125.

**Table 3:** Differences in BIA parameters according to the level of adherence to the Mediterranean diet, controlled for sex.

BIA Parameters	Q1	Q2	Q3	Q4	*p*-Value (η_p_^2^)
Rz (Ω)	544.9 ± 86.4	556.2 ± 82.8	554.5 ± 70.7	552.6 ± 77.9	0.544
Xc (Ω)	55.2 ± 8.6	55.3 ± 11.9	54.3 ± 8.3	55.8 ± 8.8	0.220
FM (%)	31.1 ± 8.4	31.8 ± 8.5	30.8 ± 7.6	30.2 ± 8.5	0.016 (0.020)
FFM (%)	68.9 ± 8.4	68.2 ± 8.5	69.2 ± 7.6	69.7 ± 8.5	0.016 (0.020)
MM (%)	31.9 ± 8.4	30.0 ± 6.9	30.3 ± 7.3	32.4 ± 9.0	0.013 (0.021)
SMI	8.2 ± 1.7	7.9 ± 1.6	7.7 ± 1.5	7.8 ± 1.5	0.982
TBW (%)	50.5 ± 6.2	49.9 ± 6.3	50.8 ± 5.5	51.1 ± 6.3	0.007 (0.024)
ECW (%)	46.7 ± 4.4	47.5 ± 4.5	47.8 ± 3.5	46.9 ± 3.9	0.273
ICW (%)	53.3 ± 4.4	52.5 ± 4.5	52.2 ± 3.5	53.1 ± 3.8	0.273
PA (°)	5.9 ± 1.1	5.7 ± 1.1	5.6 ± 0.7	5.8 ± 1.0	0.354
SPA (°)	1.1 ± 1.5	1.2 ± 1.9	1.3 ± 1.4	1.3 ± 1.6	0.971

Bonferroni correction *p* < 0.0045; Rz—resistance, Xc—reactance, FM—fat mass, FFM—fat-free mass, MM—muscle mass, SMI—skeletal muscle mass index, TBW—total body water, ECW—extracellular water, ICW—intracellular water, PA—phase angle, SPA—standardised phase angle, Q1—first.

**Table 4:** Coefficients from the multiple linear regression analysis of individual MEDLIFE items affecting selected BIA parameters for females.

	B	β	*p*	r_p_	VIF
Rz					
Red meat	31.817	0.150	0.007	0.145	1.095
White meat	−26.710	−0.153	0.006	−0.149	1.090
Fruit	−21.512	−0.131	0.014	−0.133	1.005
FM (%)					
Processed meat	−2.699	−0.173	0.001	−0.176	1.004
Olive oil	−2.314	−0.134	0.011	−0.137	1.004
Limit snacking between meals	−1.580	−0.107	0.041	−0.110	1.001
SMI					
Red meat	−0.478	−0.158	0.005	−0.152	1.095
White meat	0.360	0.144	0.009	0.140	1.090
Fruit	0.283	0.121	0.023	0.122	1.005
TBW (%)					
Processed meat	1.839	0.163	0.002	0.166	1.004
Olive oil	1.749	0.140	0.008	0.143	1.005
Cereals	−1.525	−0.125	0.017	−0.128	1.001

Rz—resistance, FM—fat mass, SMI—skeletal muscle mass index, TBW—total body water.

## Data Availability

The raw data supporting the conclusions of this article will be made available by the authors upon request.
